# The genome sequence of the silver-studded blue, *Plebejus argus *(Linnaeus, 1758)

**DOI:** 10.12688/wellcomeopenres.18607.1

**Published:** 2022-12-23

**Authors:** Alex Hayward, Konrad Lohse, Dominik R. Laetsch, Roger Vila, Wellcome Sanger Institute Tree of Life programme, Wellcome Sanger Institute Scientific Operations: DNA Pipelines collective, Tree of Life Core Informatics collective, Emma Taluy, Darwin Tree of Life Consortium

**Affiliations:** 1 College of Life and Environmental Sciences, Department of Biosciences, University of Exeter, Exeter, UK; 2 Institute of Ecology and Evolution, University of Edinburgh, Edinburgh, UK; 3 Institut de Biologia Evolutiva, CSIC - Universitat Pompeu Fabra, Barcelona, Spain; 4 Sanger Institute, Cambridge, UK

**Keywords:** Plebejus argus, silver-studded blue, genome sequence, chromosomal, Lepidoptera

## Abstract

We present a genome assembly from an individual male *Plebejus argus *(silver-studded blue; Arthropoda; Insecta; Lepidoptera; Lycaenidae). The genome sequence is 382 megabases in span. The entire assembly (100%) is scaffolded into 23 chromosomal pseudomolecules with the Z sex chromosome assembled. The complete mitochondrial genome was also assembled and is 27.4 kilobases in length. Gene annotation of this assembly on Ensembl identified 12,693 protein coding genes.

## Species taxonomy

Eukaryota; Metazoa; Ecdysozoa; Arthropoda; Hexapoda; Insecta; Pterygota; Neoptera; Endopterygota; Lepidoptera; Glossata; Ditrysia; Papilionoidea; Lycaenidae; Polyommatinae; Polyommatini; *Plebejus*; *Plebejus argus* (Linnaeus, 1758) (NCBI:txid242267).

## Background

The silver-studded blue butterfly, *Plebejus argus* (Linnaeus, 1758), belongs to the Lycaenidae family. The species is distributed across the Palearctic, including the UK, where it has declined in numbers considerably over the last 100 years ( Harris, 2008). The butterfly derives its name from the submarginal row of silvery-blue ‘studs’ present on the underside of the hindwing. In males, the upper side of the wings is blue with black borders, whilst in females it is brown with a row of submarginal orange spots.

The silver-studded blue is mainly found on coastal heathland, mossland, grassland, and dunes in the UK ( Thomas, 1993), but across its range it can be found also in mountainous areas, up to alpine habitats. The silver-studded blue is a poor disperser, with few individuals travelling further than 20-50 m daily ( Hovestadt & Nieminen, 2009; Ravenscroft, 1990; Sielezniew *et al.*, 2011), hindering movement of the species to new habitats.

The silver-studded blue seeks relatively warm environments in the northern part of its distribution range. UK populations are mainly restricted to the south and east of England and Wales, and it is considered extinct in the North ( Thomas, 1993). However, where populations do occur, these often consist of large numbers of individuals. While *P. argus* is considered a species of Least Concern according to the IUCN Red List for Europe ( van Swaay *et al.*, 2010), it is listed as vulnerable on the UK Red List ( Fox *et al.*, 2022). Factors contributing to its decline in the UK include habitat loss and fragmentation arising from urbanisation, agriculture and habitat succession ( Brookes *et al.*, 1997; Thomas, 1993; Thomas *et al.*, 1999).

The silver-studded blue is mostly univoltine, except for some populations that may have more than a generation every year. The caterpillars are polyphagous, and a notable variety of host plants have been recorded. Additionally, *P. argus* has an obligate mutualistic relationship with ants from the genus *Lasius*, which safeguard its eggs (which are laid close to *Lasius* ant nests) and tend its caterpillars, protecting them from parasites and predators, in return for a sugary secretion produced by the caterpillar. The caterpillars are nocturnal and spend the day protected inside the ant nest, where they pupate ( Jordano *et al.*, 1992; Ravenscroft, 1990; Seymour *et al.*, 2003; Thomas, 1993). The requirement for the presence of their ant mutualists further limits the habitats that the silver-studded blue can colonise. Furthermore, the species will seldom recolonise habitats if the distance between habitat fragments becomes too large, owing to its poor capacity for dispersal ( Ravenscroft, 1990; Thomas, 1993).

*P. argus* has 23 chromosome pairs ( Lorković, 1941). The genome sequence of the silver-studded blue may help to enhance understanding of its genetic diversity and population biology, and ultimately assist with conservation efforts ( Brookes *et al.*, 1997). In particular, there are many recorded subspecies of the silver-studded blue, and population genetic studies using genome re-sequencing data may help to resolve the validity and relationships among these.

## Genome sequence report

**Figure 1.**
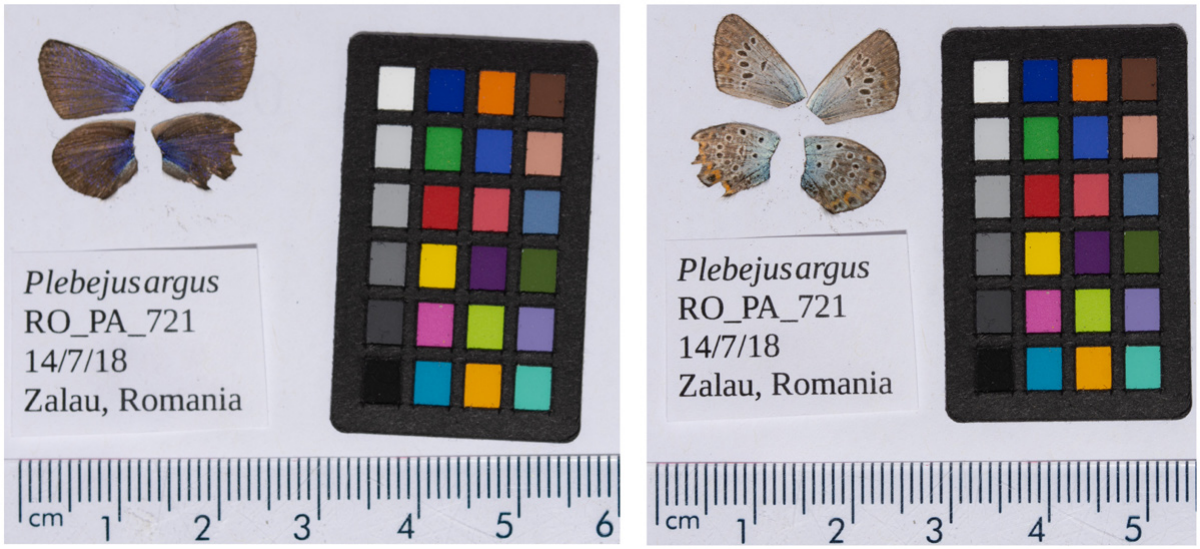
Forewings and hindwings of the male *Plebejus argus* specimen from which the genome was sequenced. Dorsal (left) and ventral (right) surface view of wings from specimen RO_PA_721 (ilPleArgu1) from Românași, Zalău, Romania, used to generate Pacific Biosciences, 10X genomics and Hi-C data.

The genome was sequenced from a single male *P. argus* ( Figure 1) collected from Românași, Zalău, Romania. A total of 23-fold coverage in Pacific Biosciences single-molecule HiFi long reads and 109-fold coverage in 10X Genomics read clouds were generated. Primary assembly contigs were scaffolded with chromosome conformation Hi-C data. Manual assembly curation corrected 182 missing/misjoins and removed 26 haplotypic duplications, reducing the assembly size by 2.31% and the scaffold number by 66.38%, and increasing the scaffold N50 by 18.92%.

**Figure 2.**
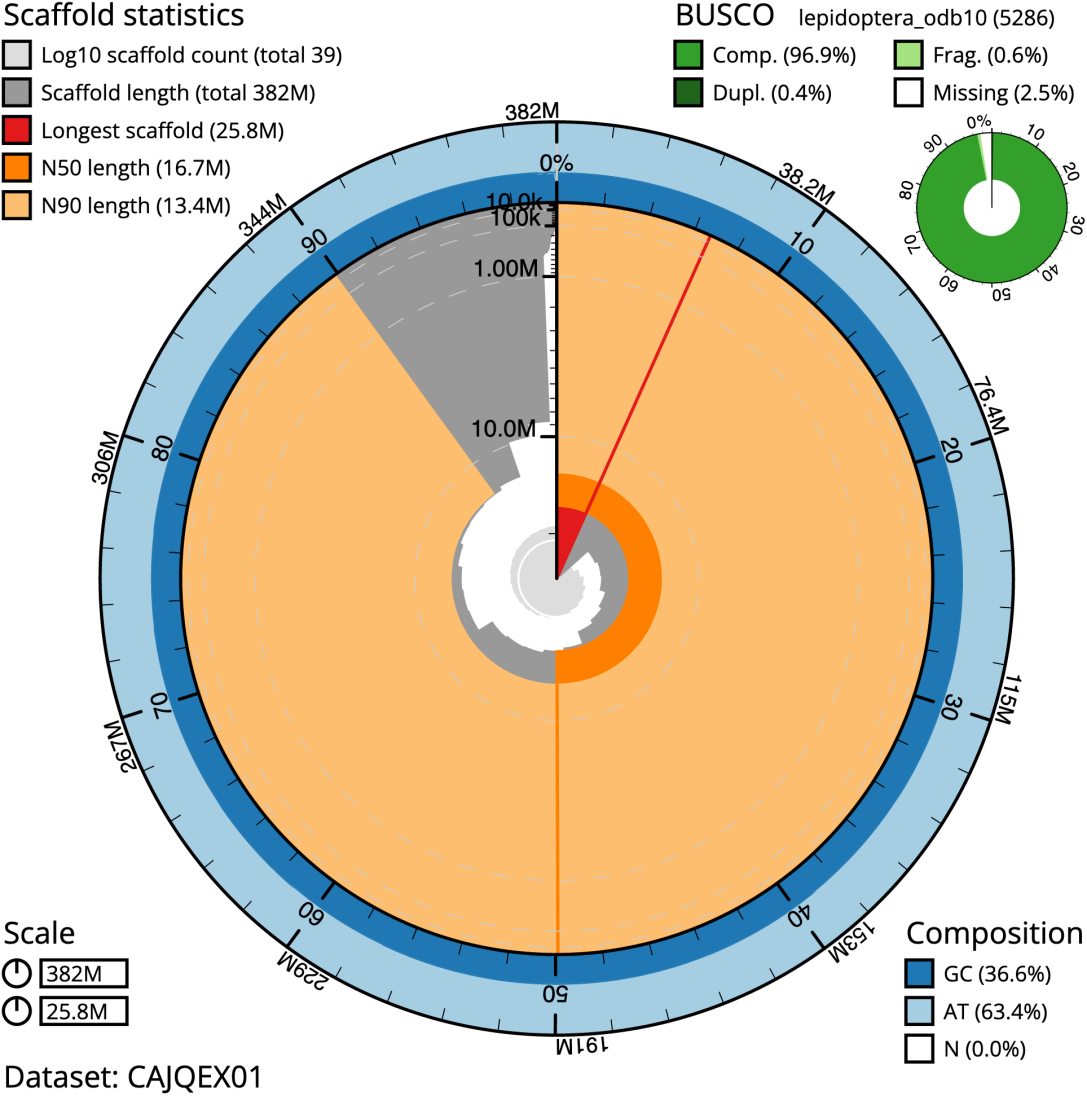
Genome assembly of *P. argus*, ilPleArgu1.1: metrics. The BlobToolKit Snailplot shows N50 metrics and BUSCO gene completeness. The main plot is divided into 1,000 size-ordered bins around the circumference with each bin representing 0.1% of the 382,108,379 bp assembly. The distribution of chromosome lengths is shown in dark grey with the plot radius scaled to the longest chromosome present in the assembly (25,784,602 bp, shown in red). Orange and pale-orange arcs show the N50 and N90 chromosome lengths (16,721,534 and 13,381,465 bp), respectively. The pale grey spiral shows the cumulative chromosome count on a log scale with white scale lines showing successive orders of magnitude. The blue and pale-blue area around the outside of the plot shows the distribution of GC, AT and N percentages in the same bins as the inner plot. A summary of complete, fragmented, duplicated and missing BUSCO genes in the lepidoptera_odb10 set is shown in the top right. An interactive version of this figure is available at https://blobtoolkit.genomehubs.org/view/ilPleArgu1.1/dataset/CAJQEX01/snail.

The final assembly has a total length of 382 Mb in 38 sequence scaffolds with a scaffold N50 of 16.7 Mb ( Table 1). The assembled sequence (100%) was assigned to 23 chromosomal-level scaffolds, representing 22 autosomes (numbered by sequence length) and the Z sex chromosome ( Figure 2– Figure 5; Table 2).

**Table 1.** Genome data for *P. argus*, ilPleArgu1.2.

*Project accession data*	
Assembly identifier	ilPleArgu1.2
Species	*Plebejus argus*
Specimen	ilPleArgu1 (genome assembly, Hi-C); ilPleArgu2 (RNA-Seq)
NCBI taxonomy ID	242267
BioProject	PRJEB43805
BioSample ID	SAMEA7523294
Isolate information	Male, whole organism (ilPleArgu1, ilPleArgu2)
*Raw data accessions*	
PacificBiosciences SEQUEL II	ERR6606789
10X Genomics Illumina	ERR6054651–ERR6054654
Hi-C Illumina	ERR6054655
PolyA RNA-Seq Illumina	ERR6054656
*Genome assembly*	
Assembly accession	GCA_905404155.2
*Accession of alternate haplotype*	GCA_905404165.1
Span (Mb)	382
Number of contigs	207
Contig N50 length (Mb)	3.7
Number of scaffolds	38
Scaffold N50 length (Mb)	16.7
Longest scaffold (Mb)	25.8
BUSCO * genome score	C:96.9%[S:96.5%,D:0.4%],F:0.6%,M:2.5%,n:5,286
*Genome annotation*	
Number of protein-coding genes	12,693

*BUSCO scores based on the lepidoptera_odb10 BUSCO set using v5.3.2. C = complete [S = single copy, D = duplicated], F = fragmented, M = missing, n = number of orthologues in comparison. A full set of BUSCO scores is available at https://blobtoolkit.genomehubs.org/view/ilPleArgu1.1/dataset/CAJQEX01/busco.

**Figure 3.**
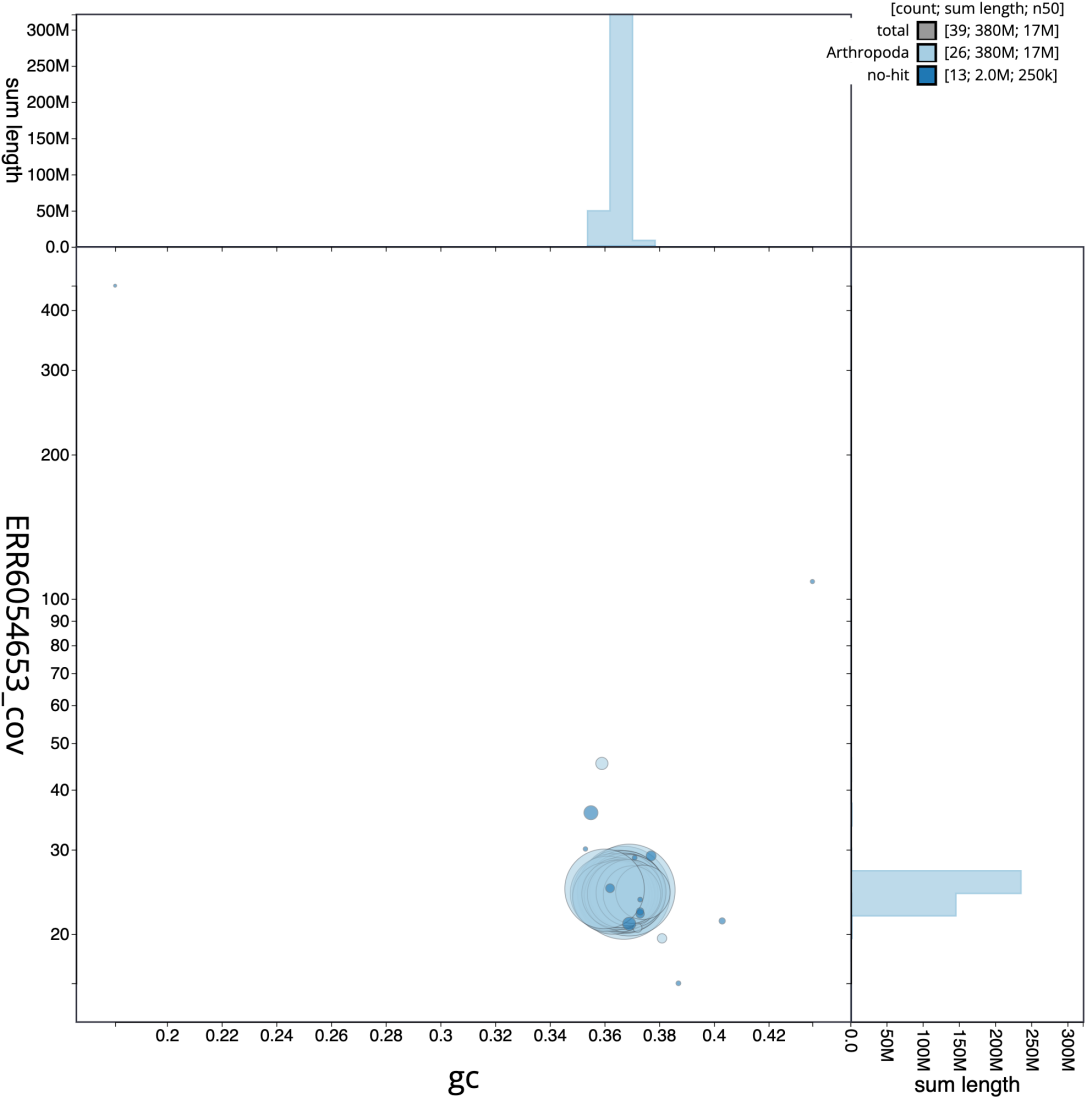
Genome assembly of *Plebejus argus*, ilPleArgu1.1: GC coverage. BlobToolKit GC-coverage plot. Scaffolds are coloured by phylum. Circles are sized in proportion to scaffold length. Histograms show the distribution of scaffold length sum along each axis. An interactive version of this figure is available at https://blobtoolkit.genomehubs.org/view/ilPleArgu1.1/dataset/CAJQEX01/blob.

**Figure 4.**
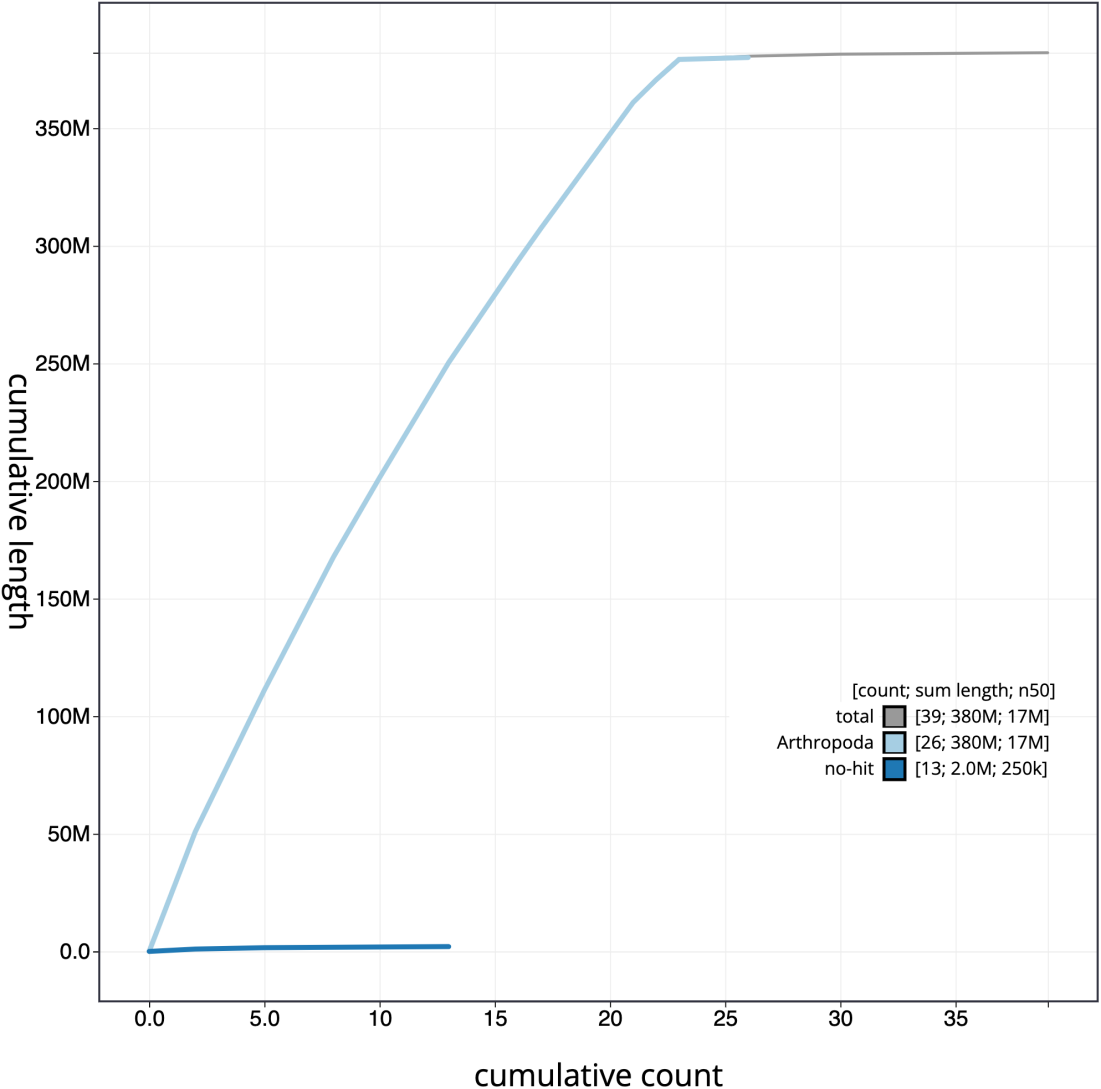
Genome assembly of *Plebejus argus*, ilPleArgu1.1: cumulative sequence. BlobToolKit cumulative sequence plot. The grey line shows cumulative length for all scaffolds. Coloured lines show cumulative lengths of scaffolds assigned to each phylum using the buscogenes taxrule. An interactive version of this figure is available at https://blobtoolkit.genomehubs.org/view/ilPleArgu1.1/dataset/CAJQEX01/cumulative.

**Figure 5.**
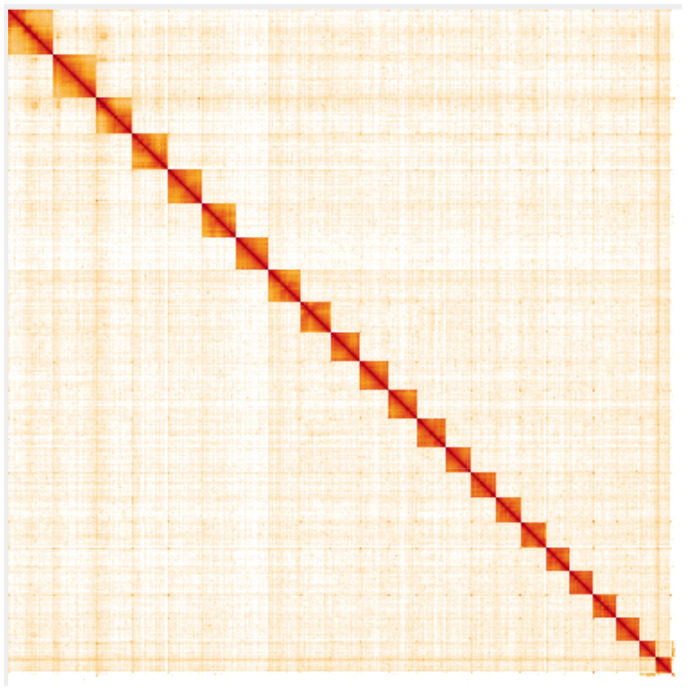
Genome assembly of *Plebejus argus*, ilPleArgu1.2: Hi-C contact map. Hi-C contact map of the ilPleArgu1.2 assembly, visualised in HiGlass. Chromosomes are arranged in size order from left to right and top to bottom. The interactive Hi-C map can be viewed at https://genome-note-higlass.tol.sanger.ac.uk/l/?d=JU9w_iyARvepzOOGpR0BQA.

**Table 2.** Chromosomal pseudomolecules in the genome assembly of *P. argus*, ilPleArgu1.2.

INSDC accession	Chromosome	Size (Mb)	GC%
FR989926.1	1	25.78	36.7
FR989927.1	2	25.11	36.9
FR989928.1	3	20.34	36.7
FR989929.1	4	20.01	36.5
FR989930.1	5	19.93	36.7
FR989931.1	6	19.24	36.3
FR989933.1	7	18.46	36.8
FR989934.1	8	17.02	36.7
FR989935.1	9	16.72	36.7
FR989936.1	10	16.42	36.6
FR989937.1	11	16.34	36.1
FR989938.1	12	16.16	36.9
FR989939.1	13	14.49	36.5
FR989940.1	14	14.38	36.6
FR989941.1	15	14.28	36.1
FR989942.1	16	13.86	36.7
FR989943.1	17	13.56	36.5
FR989944.1	18	13.5	36.4
FR989945.1	19	13.38	36.6
FR989946.1	20	13.02	36.9
FR989947.1	21	9.72	37
FR989948.1	22	8.68	37.4
FR989932.1	Z	18.84	36
FR989949.2	MT	0.03	36.3
-	Unplaced	2.84	36.9

The assembly has a BUSCO v5.3.2 ( Manni *et al.*, 2021) completeness of 96.9% (single 96.5%, duplicated 0.4%) using the lepidoptera_odb10 reference set ( *n* = 5,286). While not fully phased, the assembly deposited is of one haplotype. Contigs corresponding to the second haplotype have also been deposited.

## Genome annotation report

The ilPleArgu1.1 genome was annotated using the Ensembl rapid annotation pipeline ( Table 1; https://rapid.ensembl.org/Plebejus_argus_GCA_905404155.1/Info/Index). The resulting annotation includes 24,356 gene transcripts from 12,693 protein-coding and 3,286 non-coding genes.

## Methods

### Sample acquisition and nucleic acid extraction

A single male *P. argus* specimen (ilPleArgu1; genome assembly, Hi-C) was collected using a handnet from Românași, Zalău, Romania (latitude 47.119, longitude 23.165) by Alex Hayward (University of Exeter), Konrad Lohse, Dominik Laetsch (University of Edinburgh) and Roger Vila (Institut de Biologia Evolutiva, Barcelona). The specimen was identified by Roger Vila and snap-frozen from live in a dry shipper. A second male *P. argus* specimen (ilPleArgu2; RNA-Seq) was collected using a net from Alba, Romania (latitude 46.416051, longitude 23.192183) by Konrad Lohse, Dominik Laetsch (University of Edinburgh), Alex Hayward (University of Exeter) and Roger Vila (Institut de Biologia Evolutiva, Barcelona). The specimen was identified by Roger Vila and flash-frozen from live in a dry shipper. 

DNA was extracted at the Scientific Operations Core, Wellcome Sanger Institute. The ilPleArgu1 sample was weighed and dissected on dry ice with tissue set aside for Hi-C sequencing. Whole organism tissue was disrupted by manual grinding with a disposable pestle. Fragment size analysis of 0.01–0.5 ng of DNA was then performed using an Agilent FemtoPulse. High molecular weight (HMW) DNA was extracted using the Qiagen MagAttract HMW DNA extraction kit. Low molecular weight DNA was removed from a 200-ng aliquot of extracted DNA using 0.8X AMpure XP purification kit prior to 10X Chromium sequencing; a minimum of 50 ng DNA was submitted for 10X sequencing. HMW DNA was sheared into an average fragment size of 12–20 kb in a Megaruptor 3 system with speed setting 30. Sheared DNA was purified by solid-phase reversible immobilisation using AMPure PB beads with a 1.8X ratio of beads to sample to remove the shorter fragments and concentrate the DNA sample. The concentration of the sheared and purified DNA was assessed using a Nanodrop spectrophotometer and Qubit Fluorometer and Qubit dsDNA High Sensitivity Assay kit. Fragment size distribution was evaluated by running the sample on the FemtoPulse system.

RNA was extracted from whole organism tissue of ilPleArgu2 in the Tree of Life Laboratory at the WSI using TRIzol, according to the manufacturer’s instructions. RNA was then eluted in 50 μl RNAse-free water and its concentration RNA assessed using a Nanodrop spectrophotometer and Qubit Fluorometer using the Qubit RNA Broad-Range (BR) Assay kit. Analysis of the integrity of the RNA was done using Agilent RNA 6000 Pico Kit and Eukaryotic Total RNA assay.

### Sequencing

Pacific Biosciences HiFi circular consensus and 10X Genomics Chromium read cloud sequencing libraries were constructed according to the manufacturers’ instructions. Sequencing was performed by the Scientific Operations core at the Wellcome Sanger Institute on Pacific Biosciences SEQUEL II (HiFi), Illumina HiSeq 10X and Illumina HiSeq 4000 (RNA-Seq) instruments. Hi-C data were generated in the Tree of Life laboratory from remaining whole organism tissue of ilPleArgu1 using the Arima v1 kit and sequenced on a HiSeq 10X instrument. 

### Genome assembly

Assembly was carried out with Hifiasm ( Cheng *et al.*, 2021) and haplotypic duplication was identified and removed with purge_dups ( Guan *et al.*, 2020). One round of polishing was performed by aligning 10X Genomics read data to the assembly with longranger align, calling variants with freebayes ( Garrison & Marth, 2012). The assembly was then scaffolded with Hi-C data ( Rao *et al.*, 2014) using SALSA2 ( Ghurye *et al.*, 2019). The assembly was checked for contamination and corrected using the gEVAL system ( Chow *et al.*, 2016) as described previously ( Howe *et al.*, 2021). Manual curation ( Howe *et al.*, 2021) was performed using gEVAL, HiGlass ( Kerpedjiev *et al.*, 2018) and Pretext ( Harry, 2022). The mitochondrial genome was assembled using MitoHiFi ( Uliano-Silva *et al.*, 2021), which performs annotation using MitoFinder ( Allio *et al.*, 2020). The genome was analysed and BUSCO scores were generated within the BlobToolKit environment ( Challis *et al.*, 2020). Table 3 contains a list of all software tool versions used, where appropriate.

**Table 3.** Software tools used.

Software tool	Version	Source
BlobToolKit	3.2.6	Challis *et al.*, 2020
freebayes	1.3.1-17-gaa2ace8	Garrison & Marth, 2012
gEVAL	N/A	Chow *et al.*, 2016
Hifiasm	0.15.3	Cheng *et al.*, 2021
HiGlass	1.11.6	Kerpedjiev *et al.*, 2018
longranger align	2.2.2	https://support.10xgenomics.com/genome-exome/software/pipelines/latest/advanced/other-pipelines
MitoHiFi	2	Uliano-Silva *et al.*, 2021
PretextView	0.2.x	https://github.com/wtsi-hpag/PretextView
purge_dups	1.2.3	Guan *et al.*, 2020
SALSA2	2.2	Ghurye *et al.*, 2019

### Genome annotation

The Ensembl gene annotation system ( Aken *et al.*, 2016) was used to generate annotation for the *Plebejus argus* assembly (GCA_905404155.1). Annotation was created primarily through alignment of transcriptomic data to the genome, with gap filling via protein-to-genome alignments of a select set of proteins from UniProt ( UniProt Consortium, 2019).

## Data Availability

European Nucleotide Archive: *Plebejus argus* (silver-studded blue). Accession number PRJEB43805; https://identifiers.org/ena.embl/PRJEB43805 ( Wellcome Sanger Institute, 2022). The genome sequence is released openly for reuse. The *P. argus* genome sequencing initiative is part of the Darwin Tree of Life (DToL) project. All raw sequence data and the assembly have been deposited in INSDC databases. Raw data and assembly accession identifiers are reported in Table 1.

## Author information

Members of the Wellcome Sanger Institute Tree of Life programme are listed here: https://doi.org/10.5281/zenodo.4783585.

Members of Wellcome Sanger Institute Scientific Operations: DNA Pipelines collective are listed here: https://doi.org/10.5281/zenodo.4790455.

Members of the Tree of Life Core Informatics collective are listed here: https://doi.org/10.5281/zenodo.5013541.

Members of the Darwin Tree of Life Consortium are listed here: https://doi.org/10.5281/zenodo.4783558.
