# Supplementary material for: The genome sequence of the silver-studded blue, Plebejus argus (Linnaeus, 1758)
Source: Wellcome Open Res. 2022 Dec 23;7:315. [Version 1] doi: 10.12688/wellcomeopenres.18607.1 (PMC9975408; doi:10.12688/wellcomeopenres.18607.1)
Supplement: Peer review history

# Peer review: Reviewer response for version 1

**DOI:** 10.21956/wellcomeopenres.20634.r53807

**Published:** 2023-01-13

**Authors:** Bin Liang

**Affiliations:** 1 College of Life Science, University of Inner Mongolia, Hohhot, China

This paper completes high-quality genomic denovo assembly for *Plebejus argus* and combined with transcriptomic data and UniProt for genome annotation. The authors used Pacific Biosciences, Illumina HiSeq 10X and Hi-C data for mixed assembly, scaffolded into 23 chromosomal pseudomolecules with the Z sex chromosome and mitochondrial genome.

 Meanwhile, the N50 index, BUSCO gene integrity, GC coverage, cumulative sequence, Hi-C contact map and chromosome assembly information of the assembly results are displayed, reflecting the completeness and accuracy of the research results. In summary, the establishment of its high-quality genome provides an important reference genome for the genomics and ecology study of blue butterfly.

 Only one question is about the size of mitochondrial genome. In abstract, author mentioned mitogenome size is 27.4 kilobases in length. I think this is an error. Its length is about 15,390 bp（accession: FR989949.3, similar with another published report.

Are sufficient details of methods and materials provided to allow replication by others?

Yes

Is the rationale for creating the dataset(s) clearly described?

Yes

Are the datasets clearly presented in a useable and accessible format?

Yes

Are the protocols appropriate and is the work technically sound?

Yes

Reviewer Expertise:

Phylogenetic study of bird and butterfly

I confirm that I have read this submission and believe that I have an appropriate level of expertise to confirm that it is of an acceptable scientific standard.

# Peer review: Reviewer response for version 1

**DOI:** 10.21956/wellcomeopenres.20634.r53803

**Published:** 2023-01-04

**Authors:** Elena A. Pazhenkova

**Affiliations:** 1 Department of Biology, Biotechnical Faculty, University of Ljubljana, Ljubljana, Slovenia

This article presents a genome assembly for the silver-studded blue butterfly, *Plebejus argus *(Lepidoptera, Lycaenidae).

 Authors have combined data from Pacific Biosciences, Illumina HiSeq 10X and Hi-C to construct high-quality assembly, scaffolded into 23 chromosomal pseudomolecules, including the Z sex chromosome as well as mitochondrial genome. The second male individual was sequenced on Illumina HiSeq 4000 (RNA-Seq) to generate gene annotation. The inclusion of N50 metrics, GC coverage, cumulative sequence length and Hi-C contact map figures is helpful in understanding the assembly process and results. The tables providing genome data accession numbers, chromosomal assembly information and description of software used are also useful for reproducibility of the research.

 Overall, the article is an important contribution to the biodiversity studies and the reference genome provided can be a solid base for further population genomics and evolutionary research in blue butterflies.

Are sufficient details of methods and materials provided to allow replication by others?

Yes

Is the rationale for creating the dataset(s) clearly described?

Yes

Are the datasets clearly presented in a useable and accessible format?

Yes

Are the protocols appropriate and is the work technically sound?

Yes

Reviewer Expertise:

Entomology, bioinformatics, ecology

I confirm that I have read this submission and believe that I have an appropriate level of expertise to confirm that it is of an acceptable scientific standard.
